# Measuring Aqueduct of Sylvius Cerebrospinal Fluid Flow in Multiple Sclerosis Using Different Software

**DOI:** 10.3390/diagnostics11020325

**Published:** 2021-02-17

**Authors:** Maria Marcella Laganà, Dejan Jakimovski, Niels Bergsland, Michael G. Dwyer, Francesca Baglio, Robert Zivadinov

**Affiliations:** 1 IRCCS, Fondazione Don Carlo Gnocchi ONLUS, 20148 Milan, Italy; npbergsland@bnac.net (N.B.); fbaglio@dongnocchi.it (F.B.); 2 Buffalo Neuroimaging Analysis Center (BNAC), Department of Neurology, Jacobs School of Medicine and Biomedical Sciences, University at Buffalo, State University of New York, Buffalo, NY 14203, USA; djakimovski@bnac.net (D.J.); mgdwyer@bnac.net (M.G.D.); rzivadinov@bnac.net (R.Z.); 3 Center for Biomedical Imaging at Clinical Translational Science Institute, University at Buffalo, State University of New York, Buffalo, NY 14203, USA

**Keywords:** magnetic resonance imaging, phase contrast MRI, cerebrospinal fluid, aqueduct of Sylvius, multiple sclerosis

## Abstract

Aqueduct of Sylvius (AoS) cerebrospinal fluid flow can be quantified using phase-contrast (PC) Magnetic Resonance Imaging. The software used for AoS segmentation might affect the PC-derived measures. We analyzed AoS PC data of 30 people with multiple sclerosis and 19 normal controls using three software packages, and estimated cross-sectional area (CSA), average and highest AoS velocity (Vmean and Vmax), flow rate and volume. Our aims were to assess the repeatability and reproducibility of each PC-derived measure obtained with the various software packages, including in terms of group differentiation. All the variables had good repeatability, except the average Vmean, flow rate and volume obtained with one software package. Substantial to perfect agreement was seen when evaluating the overlap between the AoS segmentations obtained with different software packages. No variable was significantly different between software packages, with the exception of Vmean diastolic peak and CSA. Vmax diastolic peak differentiated groups, regardless of the software package. In conclusion, a clinical study should preliminarily evaluate the repeatability in order to interpret its findings. Vmax seemed to be a repeatable and reproducible measure, since the pixel with its value is usually located in the center of the AoS, and is thus unlikely be affected by ROI size.

## 1. Introduction

The cerebrospinal fluid (CSF) has various fundamental vital functions: it acts as a buffer, protecting the brain during head trauma, supports the brain weight, and helps maintaining stable central nervous system temperature [1]. With its complex circulation, the CSF provides nutrition, waste removal [2], maintains stable intracranial pressure after the intracranial volumetric increment during systole [3,4].

CSF flow can be noninvasively quantified using cardiac-gated cine phase contrast (PC) Magnetic Resonance Imaging (MRI) [5]. 2D-PC, quantifying the flow passing through an acquisition plane, is routinely used for examining the Aqueduct of Sylvius (AoS) CSF hydrodynamics in people with normal pressure hydrocephalus undergoing shunt placement procedures [6], and in subjects with aqueductal stenosis [7]. Measures derived from AoS PC-MRI provide indirect information on intracranial pressure [8]. Related to this, increased aqueductal CSF pulsatility has been shown to be related to dirty appearing white matter areas in a group of subjects without neurological diseases [9]. Hyperdynamic AoS CSF and enlarged AoS cross-sectional area (CSA) have also been shown in people with multiple sclerosis (pwMS) [10,11,12,13], but not with Alzheimer’s disease and mild cognitive impairment [14,15]. Lower net CSF flow volume has been reported in pwMS compared to normal controls (NC), with a significant association to impaired venous outflow [16]. AoS flow and CSA changes over time have also recently been reported [13] in pwMS.

Despite being used for a long time and in numerous clinical cases, the AoS CSF flow measures derived from PC-MRI have various sources of variability that should be taken into account when a clinical study is designed. The main sources of variability are summarized in the following.

First, the magnetic field strength affects the image quality, because higher field strength has a better signal-to-noise ratio (SNR). The SNR effect on flow estimates has been previously investigated [17]. Even though the field strength does not seem to affect the precision and accuracy of the PC-MRI flow quantification [17], higher temporal and spatial resolutions can be achieved with greater field strengths [18]. The latter is crucial for AoS CSF flow quantification, since the AoS is narrow, and its limited spatial resolution causes diameter dependent systematic overestimations in CSF pulsatile volume changes [19]. Moreover, increasing the SNR is important because the AoS CSF flow signal is low due to slow CSF velocity when compared to that of arterial and venous blood flow [17].

Second, different sequence parameters might cause different absolute flow values. Since the spatial resolution impacts the partial volume effect between brain parenchyma and AoS CSF, the effect of different resolutions was assessed by previous studies with simulations [20], and in vivo [18]. The latter study used 7T MRI, and showed CSA overestimation and velocity underestimation for the sequence with the lowest spatial resolution, due to the higher partial volume effect of static tissue included in the AoS pixels.

Third, different approaches have been used in the literature for drawing the regions of interest (ROI) corresponding to the AoS and to the static tissue. With regard to the latter, the effect of different positioning has been previously investigated [21], and similar strategies are suggested in literature [18]. Lee and colleagues [21] showed that positioning the baseline region anteriorly (in the midbrain) or laterally (in the temporal lobe) to the AoS did not affect the estimates of peak velocity and mean flow rate. As to the AoS ROI drawing, since the flow measures might depend on how the ROI is drawn, some publications reported the intra- or inter-operator variability [14,18,19,22]. However, despite various methods and software packages used in the literature, to the best of our knowledge, the effect of using different commercially available software packages on AoS CSF flow measures has never been investigated. For this reason, our study focused on this aspect, as detailed in our study aims.

The various methods of AoS contour drawing used in the literature can be manual [12,19,23,24], semiautomatic [11,25], or automatic [24,26,27,28]. The latter methods evaluate the velocity over time, finding those voxels where the temporal signal has the expected oscillatory shape [24], and spectral frequency components [28], or those with high temporal correlation [26,27]. Even though they have an intrinsically high repeatability, these approaches require a dedicated post-processing algorithm, and a threshold has to be defined for delimiting the AoS borders. Conversely, there are various commercially available software packages for manual or semiautomatic ROI drawing and flow computation, used in numerous AoS CSF studies [11,12,18,19,20,23,24]. These packages can be used either on scanner workstations, i.e., manufacturer’s software [12,29,30], or on separate workstations/PCs [18]. The ROI size and position might change the estimated CSF velocity [22,29] since the inclusion of parenchyma (static voxels) in the AoS contours decreases the average estimated velocity. Nonetheless, manual and semiautomatic AoS segmentations are still widely used in clinical studies [14,18,19,22].

Against this background, we performed a methodological study using three different commercially available software packages for PC-MRI flow quantification. They allow drawing of the AoS ROI with different semiautomatic methods, and to compute various AoS CSF flow parameters. The preliminary aim was to assess the repeatability of each software package. Then, we aimed to evaluate the reproducibility of each measure of interest, testing if the various measures were different depending on the software, and finally if the software choice could influence the differentiation between pwMS and NC.

## 2. Materials and Methods 

### 2.1. Study Population

The subjects included in this study were pwMS and NC, randomly chosen from a previously published clinical study of cardiovascular, environmental and genetic factors in pwMS [13]. The inclusion criteria were the following: (1) age between 18 and 75 years; (2) availability of a AoS cine PC imaging; (3) diagnosis with the 2010-revised McDonald criteria for the patients [31]. The exclusion criteria included: (1) pregnant or nursing mothers; (2) presence of congenital malformations that affect the cerebrospinal fluid anatomy (e.g., Chiari malformations, congenital hydrocephalus); (3) no clinically defined relapse or use of intravenous corticosteroid within 30 days of the MRI examination, for the NC no presence of current nor history of past major neurological disorder.

The pwMS were clinically evaluated by an experienced neurologist and were characterized by the Expanded Disability Status Scale (EDSS) scores [32]. Other recorded clinical parameters include disease duration, the type of disease modifying therapy, and MS phenotype.

### 2.2. MRI Acquisition and Processing

All the MRI exams were performed with a 3T GE Signa Excite HD 12 Twin Speed 8-channel scanner (General Electric, Milwaukee, WI, USA) and an 8-channel head and neck coil. 

**Figure 1:**
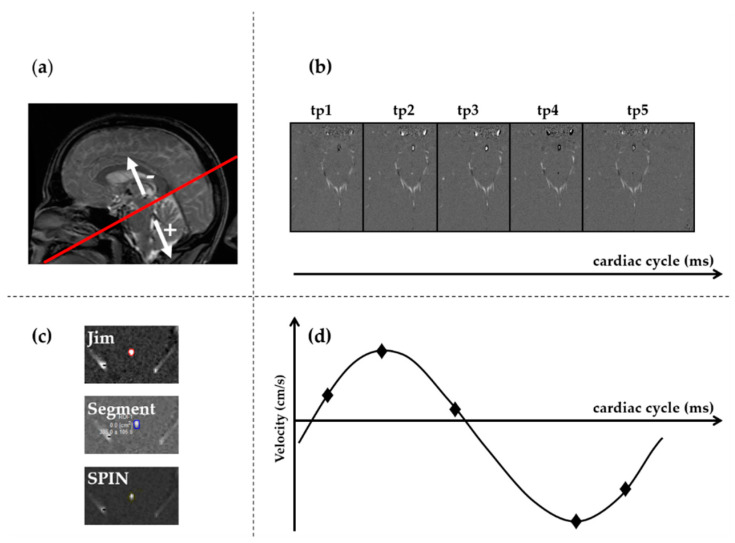
Phase-contrast (PC) MRI of the aqueduct of Sylvius (AoS): acquisition and processing. The acquisition plane was positioned on the sagittal localizer, as displayed by the red line in (**a**). The phase image sign convention is schematized in (**a**): the positive flow is that through the plane in the caudal direction, i.e., to the fourth ventricle, during the systolic phase. In (**b**), examples of phase images at 5 of the 32 time points (tp) are shown: caudal flows are visible as bright pixels in the phases of time points tp1, tp2, and tp3. Cranial flows are visible as dark pixels in the phase images of the tp4, and tp5. The time point with the highest flow-parenchyma contrast is show in (**c**), where the AoS segmentations using the three software are shown. A schematic of typical cerebrospinal fluid (CSF) velocity of any pixel inside the AoS across the normalized cardiac cycle is represented in (**d**), where the velocities of the five time points are represented.

The AoS CSF was evaluated using a single slice cine 2D PC sequence, with a pulse oximeter-gated procedure for obtaining 32 points of the cardiac cycle with a velocity encoding of 20 cm/s. The PC sequence had the following parameters: echo time (TE) = 7.9 ms, repetition time (TR) = 40 ms, flip angle = 20°, slice thickness = 4 mm, matrix = 256 × 256, FOV = 10.0 cm. We chose such an axial resolution (0.39 × 0.39 mm^2^) in order to have at least four pixels covering the AoS diameter, for good flow quantification, as suggested in [19,20]. The imaging plane was positioned at the level of the ampulla, perpendicularly to the AoS axis, using the midsagittal scout as reference [21] (Figure 1A). According to the manufacturer’s convention, CSF flow directed towards the third ventricle (during diastole) was negative, and vice versa the flow directed towards the fourth ventricle (during systole) was positive (Figure 1).

The PC cine images were processed in a blind manner, after their quality was evaluated and confirmed. Three different software programs were used for the processing: (1) Java Image Manipulation tool—Jim version 8.0, Xinapse Systems, Leicester, UK (http://www.xinapse.com/ accessed on 8 December 2020); (2) Segment version 2.2 R6887 (MedViso, Lund, Sweden—http://segment.heiberg.se accessed on 8 December 2020) [33], freely available for research purposes, which was recently used for processing PC-MRI of the AoS CSF [18]; (3) signal processing in NMR (SPIN) software (SpinTech Inc, Bingham Farms, MI, USA) [20], with a free license as research collaborators.

A single trained operator used each software package to compute various measures of the AoS CSF flow, using the following steps.

**Figure 2:**
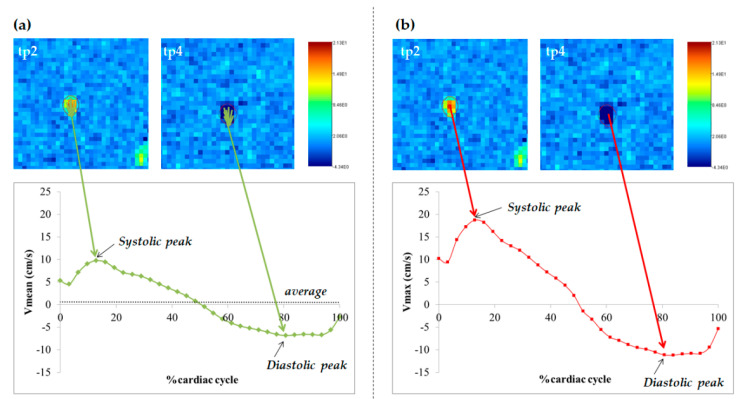
Aqueduct of Sylvius (AoS) cerebrospinal fluid (CSF) velocity parameters of interest. Phase images corresponding to the time points (tp) tp2 and tp4 of Figure 1, representing the systolic and diastolic peaks, are shown in the upper part of (**a**) and (**b**). In (**a**), for each time point, the velocities of all the pixels inside the segmented AoS are averaged (Vmean). The Vmean time course over the normalized [28] cardiac cycle is shown at the bottom. In (**b**), the pixel with the highest velocity (Vmax) is selected for each tp, and its time course over the normalized cardiac cycle is shown at the bottom. The parameters of interest used in our statistical analyses are written in italic.

**Figure 3:**
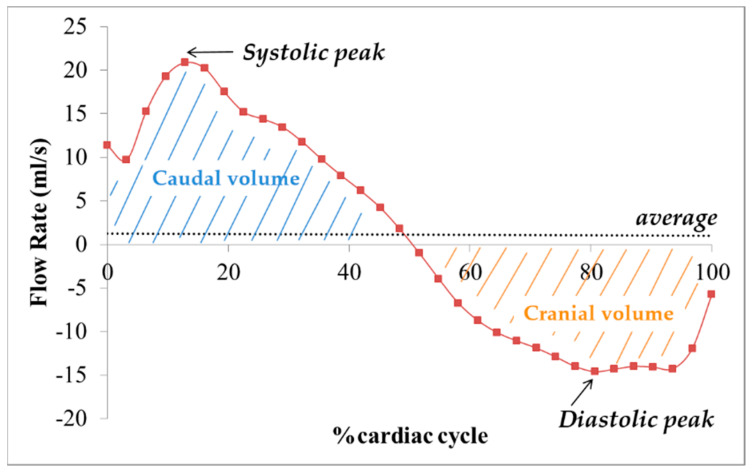
Aqueduct of Sylvius (AoS) cerebrospinal fluid (CSF) flow rate and volume. The flow rate (Vmean*AoS CSA) time course over the normalized [28] cardiac cycle is shown. The caudal and cranial volumes are schematically represented, as the area under the curve of the flow rate to the fourth and third ventricle, respectively. In our study, we considered the average flow rate, its systolic and diastolic peaks, the caudal and cranial volumes and their difference (net flow volume).

1. The time frame with the highest flow, i.e., with the highest contrast of CSF from the surrounding parenchyma, was visually selected (Figure 1B).
2. The images were magnified (Figure 1C) so that the AoS was easily visible on the screen, and two kinds of regions of interest (ROIs) were drawn: one corresponding to AoS contour and another one in an area of static tissue (NFA: no-flow area) (Supplementary Figure S1). The latter was used as a reference for correcting the phase background and was manually drawn anteriorly to the AoS [18,21]. The former was drawn semiautomatically or manually in different ways, depending on the software (Figure 1C). In particular, with Jim, we used its semiautomated local thresholding technique, which detects the contours after a pixel of the border is manually identified, as usually done in MS studies for semiautomatic lesion contour drawing [34]. With Segment we manually drew the AoS ROI and then we used the “Refine ROI” tool [33]. With SPIN, we used the region growing approach [20], which requires an initialization with the manual identification of a pixel inside the AoS. All the ROIs were copied to all the time frames. If necessary, a manual adjustment could be performed with all the software packages.
3. The velocity, corrected for the phase offset, was computed for each pixel inside the AoS ROI and for each frame of the cardiac cycle (Figure 1D) using each software package. In particular, the velocity was corrected for background velocity by subtracting the average value inside the NFA. The effect of this correction on the mean AoS velocity over the cardiac cycle is shown in Supplementary Figure S2 for one subject, for each software package.
4. The following measures were computed for each time frame of the cardiac cycle: (1) the cross-sectional area (CSA, in mm^2^) of the AoS; (2) mean velocity (Vmean) in cm/s (Figure 2A), as the spatially averaged velocity inside the segmented AoS (sum of all the velocities inside the AoS, divided by the AoS CSA); (3) maximal velocity (Vmax) in cm/s (Figure 2B), as the velocity with the highest value among all the velocities inside the segmented AoS; (4) flow rate (Vmean*AoS CSA) in mL/s (Figure 3). The following measures were computed and retained in the statistical analyses (represented and written in italic in Figure 2 and Figure 3): the average over the cardiac cycle of CSA, Vmean, Vmax, flow rate; the systolic and diastolic peaks of Vmax and Vmean. Moreover, the volumes displaced during the systolic and diastolic phases, i.e., the caudal and cranial volumes, were computed by integrating over time the flow rate to the fourth and third ventricle respectively. The net flow volume was the difference between the two last volumes (considered as absolute measures).

### 2.3. Statistical Analysis

All statistical analyses were performed in SPSS 25.0 (IBM Corp. Armonk, NY, USA).

The normality distribution of the data was determined by Kolmogorov-Smirnov test.

The demographic differences between MS and NCs were derived by parametric (χ^2^ test, Student’s *t*-test) and non-parametric (Mann-Whitney U-test) comparisons tests, where appropriate.

Ten subjects were randomly selected and re-processed with all software packages by the same operator after 2 weeks, in order to assess the intra-rater repeatability of the AoS PC-derived measures. For each measure of interest (Figure 2 and Figure 3) and each software package, the intraclass correlation coefficient (ICC) was computed from the two subsequent measures. An ICC value > 0.90 was classified as excellent, 0.71 ≤ ICC ≤ 0.90 good, 0.51 ≤ ICC ≤ 0.70 acceptable, 0.31 ≤ ICC ≤ 0.50 insufficient and ICC ≤ 0.30 as poor [35].

Since the various software packages segment the AoS in different ways, as explained above, we evaluated the segmentation reproducibility by assessing the degree of the spatial overlap between paired segmentations, with the Dice Similarity Coefficient (DSC).

The DSC was computed using a MATLAB script, as follows:

(1) $$DSC_{A,B}=\;\frac{2\times \left(A\cap B\right)}{A+B},$$

where *A* and *B* correspond to the masks of the segmented AoS, obtained using two software packages, and *A* ∩ *B* is the number of common voxels between them. The masks were obtained using Jim and SPIN graphical user interfaces for the respective segmentations, while an ad hoc script was used in order to read the ROI coordinates obtained using Segment, and filling the contour. The strength of the overlap according to the DSC was classified as almost perfect between 0.81 and 1; substantial between 0.61 and 0.8; moderate between 0.41 and 0.6; fair between 0.21 and 0.4; and slight between 0 and 0.2 [36]. Given that the location of similar segmentation mismatches might have different impact on the hydrodynamic measures, we evaluated their reproducibility, in addition to that of the CSA, as follows.

General linear models (GLM) were used in order to evaluate whether software and group (MS and NC) have an effect on each variable of interest, covarying for age and sex. Specifically, for each GLM, the measure of interest was the independent variable, while the software, group, age and sex were the independent variables, taking male as the reference for sex, and SPIN as the reference for software. Age and sex were taken into account, because it was recently shown that they influence many PC-derived measures in healthy subjects [23].

Another GLM covaried for age and sex, and the repeated measures analysis of variance (RM-ANOVA/mixed model) were used in order to test pairwise differences between software packages. For both the analyses, Bonferroni-corrected pair-wise post hoc comparisons were reported. Finally, separately for each software package, we tested if any of the PC-derived measures were statistically different between pwMS and HC, using a GLM covaried for age and sex. Bonferroni-corrected pair-wise post hoc comparisons were assessed.

The *p*-values and multiple comparison corrected q-values <0.05 were considered statistically significant.

## 3. Results

### 3.1. Demographic and Clinical Characteristics

The PC-MRI of 30 pwMS and 19 NC were evaluated. The demographic and clinical characteristics of the two groups of subjects are shown in Table 1. 

The pwMS and NCs were age (51.8 ± 8.8 and 48.4 ± 12.5 years old, *t*-test *p* = 0.261) and sex (13/17 and 5/14, *χ*^2^ test *p* = 0.362) matched. The MS population had an average disease duration of 17.5 years and the median EDSS was 2.5. The MS population consisted of 17 RRMS and 13 PMS patients. Nine (30.0%) MS patients were on interferon-β, eight (26.7%) on glatiramer acetate, seven (23.3%) on natalizumab, and six (20.0%) did not use any disease modifying treatment (DMT).

### 3.2. Phase Contrast Data Quality

All the PC planes were correctly positioned, the images were free of artifacts, and the phase signal was good. As such, all the subjects were processed and included in the statistical analyses.

### 3.3. Repeatability Results

Most of the measures showed good or excellent repeatability regardless of the software used, as reported in Table 2. However, with Jim, the average Vmean had only an acceptable ICC; average flow rate and net volume had a poor and not significant ICC.

### 3.4. Reproducibility Results: Differences among Software Packages and between Groups

Comparing the spatial overlap between software packages A and B, the median(range) of the DSC (DSC A–B) was as follows. DSC_Jim_Segment_ = 0.902(0.766–1); DSC_Jim_SPIN_ = 0.877(0.727–0.968); DSC_Segment_SPIN_ = 0.867(0.742–0.963). The median DSC was almost perfect for each comparison, with exception of two subjects for Jim vs. Segment comparison, and five agreement subjects for the Jim vs. SPIN and Segment vs. SPIN comparisons.

The effects of age, sex software (Jim, Segment and SPIN), and group (MS or NC) on each variable of interest are reported in Table 3. 

With regard to the demographic variables, age significantly affected the CSA, Vmax peaks, FR systolic peak, negative and positive volumes. The greater the age was, the larger these variables were (Beta > 0 for the positive variables and Beta < for the negative variables). Sex significantly affected all the PC-derived measures, with the exception of the Vmean diastolic peak (*p*-value = 0.099) and average (*p* = 0.050), with larger values for males compared to females (0 = female, 1 = male, Beta > 0 for the positive variables and Beta < for the negative variables). 

For all the PC-derived measures, software did not have a significant effect. Conversely, the group had a significant effect on the CSA, Vmax diastolic peak, Vmean diastolic peak, flow rate diastolic and systolic peaks, positive and negative volumes, with greater values for MS compared to NC.

The post hoc analyses results are shown in Table 4 for the software and in Table 5 for the groups. 

Table 4 confirms that there was no significant difference between the average measures derived from pairs of software packages, but the paired comparisons revealed a significantly different CSA between Jim and Segment and Vmean diastolic peak between Jim and SPIN. The means and standard deviations (SD) of the different measures obtained with each software package are reported in different columns.

Table 5 shows the means and SD of the different measures obtained in MS and NC subgroups, separately for the three software packages, highlighting which software was able to discriminate the two groups after Bonferroni correction. In particular, the CSA and flow rate diastolic peak survived the post hoc correction with Jim, while the Vmax diastolic peak was significantly different between MS and NC with all software packages. Conversely, the Vmean peaks, flow rate systolic peak, negative and positive volumes found from the GLM reported in Table 4 did not survive the post hoc analysis, and were not significantly different between pwMS and NC.

## 4. Discussion

We analyzed the AoS PC-MRI data of 30 pwMS and 19 NC using three different commercially available software packages, quantified the flow over the cardiac cycle, and estimated various measures of interest typically used in clinical studies. Most of them showed good repeatability when computed twice using the same software, excluding the average of Vmean, flow rate and volume obtained with Jim. The DSC comparing the AoS segmentations from different software packages showed agreement between them, with an almost perfect segmentation reproducibility for most of the cases. Our GLM analyses revealed that age, sex and diagnosis of MS had an effect on many variables, but that the software used did not have a significant effect on any variable. However, the post hoc analyses showed significantly different CSA between Jim and Segment and different Vmean diastolic peak between Jim and SPIN. Additionally, most of the PC-derived variables were unable to distinguish between pwMS and NC regardless of software package, with the exception of Vmax diastolic peak, which was significantly greater in pwMS obtained by all software. Two software-specific variables were able to discriminate between pwMS and NC: Jim-derived CSA and Flow rate diastolic peak were significantly greater in pwMS.

Globally, these findings suggest that PC-MRI of the AoS allows calculations of CSF measures of interest that are repeatable and that may provide useful clinical information regardless of which method is used. However, the testing repeatability and reproducibility of various PC-derived morphological and hydrodynamic measures made it possible to show that some are affected by ROI placement, with a potential impact on its estimation and clinical results.

The clinical utility of the current methodological study focuses on software packages that are commercially available, and that can be used by any operator with PC-MRI experience, even without the need for programming skills. Conversely, ad hoc automatic methods [24,26,27,28] have been developed, but are not openly available for the clinical practice. The automatic methods intrinsically have high repeatability; however, with this study, we also showed the high repeatability with manual or semi-automatic software packages. Standardizing the processing procedure, as discussed in the following paragraphs, can further facilitate their clinical use.

The three software packages we used for the analyses had different strategies for contour definition, as stated earlier. The drawing of the AoS contour may affect the flow estimates [20,22]; however, these effects did not influence most of the CSF measurements in a significant way. This is probably due to the experience of the operator, who took care to use the same strategy when drawing the AoS ROI. In particular, it was drawn such that all the visible flow was included in the contours, as suggested in [14,20]. Other guidelines regarding standardizing the drawing of the ROI should be followed as much as possible. 

First of all, before drawing an AoS ROI, it is suggested to select the cardiac cycle phase where the velocity is high [18], i.e., usually during systole, in order to have a high contrast between flow and surrounding stationary tissue. Regarding the ROI size, an undersized one would overestimate Vmean, because the highest velocity values are at the center of the lumen in laminar flow, and underestimate the flow rate, because some flow is missed. However, with oversized boundaries, there is the risk of including parenchyma pixels, which underestimate Vmean by including stationary tissue. Based on theoretical computations and simulations, Jiang and colleagues showed that it is better to overestimate rather than underestimate the CSA [20] when performing PC-MRI flow quantification.

It is also important to note that the pixel with the highest velocity is in the central region of the AoS, so it is very likely to find it inside any AoS ROI, if laminar flow is assumed. This is our case, because our values of maximum velocity (highest Vmax peak: less than 19 cm/s) and CSA (always below 6.6 mm^2^) yield a Reynolds number (always below 550) far below the threshold for turbulent flow, as similarly found in [37]. Thus, it is a good strategy for considering Vmax, i.e., the maximum velocity inside the ROI (Figure 2B). Vmax should not vary with different ROI size, at least at the time points where it has high values, such as in the systole and diastole. Indeed, with our GLM and RM-ANOVA analyses, we found that systolic and diastolic Vmax peaks are the same regardless of the used software, as previously reported [22], and it had excellent repeatability. In the time points where Vmax approaches zero, it might vary depending on the drawn ROI, because similar values are included in the ROI and there is not one clearly prevailing over the other. For this reason, we did not include this variable in our study.

Understanding the dependence of Vmean, Vmax and flow rate by ROI positioning and size allows to interpret the results of our repeatability and reproducibility tests. Although fully automatic segmentation methods were not used, we adopted commercially available software with various semiautomatic region drawing approaches, obtaining promising results and highlighting aspects that warrant caution. In particular, we had good and excellent repeatability for CSA, systolic and diastolic Vmax, Vmean, flow rate and volume with all the software. Even though the CSA was significantly different between Jim and Segment, further DSC investigation obtained with all the software packages showed substantial to almost perfect agreement. However, we also acknowledge that only acceptable ICC was obtained with Jim when considering the average of Vmean, and that the Vmean diastolic peak was significantly different between Jim and SPIN. This is easily explainable as being due to the variability in the manual selection of the border pixel that allows to initialize the semiautomatic segmentation. Indeed, Vmean is influenced by the ROI size, contrary to Vmax, as previously discussed. With Jim, manually selecting a different pixel of the border produces a higher/smaller contour, and consequently a lower/higher Vmean, respectively. Since the average flow rate is computed from average Vmean, and net volume is computed from the average flow rate, even worse ICC could be expected for them in this case, as we found. These variables had a better ICC with SPIN and Segment; in particular, SPIN makes it possible to segment by indicating a pixel inside the AoS, a method that is not affected by the visual definition of border pixels and so it is more repeatable. We obtained good ICC with Segment as well, probably because the manual segmentation was automatically adapted to the border with the automatic “Refine” tool. The dependence of the average Vmean, average flow rate and net volume on the ROI position and size, at least with one of the used software packages, could be due to the low values of these measures. This is due to the kind of CSF flow, which has a typical bidirectional and sinusoidal shape, as discussed in the work of Wåhlin and colleagues [19]. They found insufficient repeatability for the average flow rate (0.41), but an excellent ICC (0.96) for volume change, which we found for caudal and cranial volume using all three methods. Similarly, Luetmer and colleagues [14] reported small interobserver variability, below the variations among subjects, for the average flow rate. Conversely, Tawfik and colleagues [22] found a good ICC (0.88) for average Vmax, and excellent (0.97) ICC for the stroke volume. However, the latter does not correspond to our net volume, because it was computed as the average of the absolute positive and negative volumes, so it is the average of caudal and cranial volume, that in our study had ICC similar to those reported by Tawfik et al. [22]. Importantly, in our study we decided to evaluate the software performance in measuring all the main CSF-based parameters that had previously been reported in the literature. Our estimates of the AoS CSF flow measurements of interest agree with those reported in the literature using the same [18] or different software [11,38]. However, for a given sequence, scanner, and processing method, it is recommended to generate specific normative reference values. With regard to the selected sequence, we highlight that we used a resolution that allowed to have at least four pixels in the AoS diameter, since it was suggested that systematic errors are expected for more coarse resolutions [19,20].

Given the results we obtained when modeling the PC-derived variables using the GLM approach (Table 3), we also suggest that age and sex should be taken into account when CSF flow is compared between groups. Our findings agree with those recently reported by Sartoretti and colleagues [23]; in particular, the increment of caudal and cranial volumes with age, and the higher values for males compared to females, as well as the higher average flow rate in males compared to females.

As for our clinical results (Table 3 and Table 5), we found several measures that were significantly higher in pwMS compared to NC, including: CSA, Vmean diastolic peak, flow rate systolic and diastolic peak, caudal and cranial volumes, and Vmax diastolic peak.

When we separately evaluated data obtained with the three methods, only Vmax was reproducible, and was significantly different between pwMS and NC with all software packages. Significant post hoc differences for CSA and flow rate diastolic peak were also obtained with Jim, but not with the other software.

A limitation of this study is that all the analyses were performed by a single operator, and no inter-rater reproducibility was conducted. Future analyses should aim at determining the extent of inter-rater differences, in one or all of the aforementioned software packages.

In conclusion, the CSF flow estimated using manual and semiautomatic contour drawing from PC-MRI provides measures of interest that are repeatable. Vmax is a reliable measure regardless the ROI position and shape, being at the center of the AoS. Its diastolic peak has excellent repeatability, and is reproducible, being significantly higher in pwMS compared to NC, regardless of the software used.

## Figures and Tables

**Table 1:** Demographic and clinical characteristics of the two groups.

Demographic/Clinical Variable	MS	NC	*p*-Value
N	30	19	-
Age in years, mean ± SD	51.8 ± 8.8	48.4 ± 12.5	0.261 §
Sex (M/F)	13/17	5/14	0.362 #
Disease duration in years, mean ± SD	17.5 ± 11.0	-	-
EDSS, median (IQR)	2.5 (1.5–6.0)	-	-
RRMS/PMS	17/13		
DMT, *n* (%)		-	-
Interferon-β	9 (30.0)	-	-
Glatiramer acetate	8 (26.7)	-	-
Natalizumab	7 (23.3)	-	-
No DMT	6 (20.0)	-	-

MS—multiple sclerosis, NC—normal controls, EDSS—Expanded Disability Status Scale, DMT—disease modifying treatment, RRMS—relapsing-remitting MS, PMS—progressive MS, SD—standard deviation, IQR—interquartile range. § Student’s and # *χ*^2^ *t*-test were used. A *p*-value lower than 0.05 was considered statistically significant.

**Table 2:** Intra-class correlation coefficient (ICC) values for each variable of interest and each software package. All the ICCs are significant, with the exception of the average flow rate and net volume computed with Jim. The *p*-values different from *p* < 0.001 are reported.

Variable of Interest		ICC Values (*n* = 10)		
		Jim	Segment	SPIN
CSA	average	0.884	0.889	0.823
Vmean	systolic peak	0.699	0.985	0.948
	diastolic peak	0.954	0.954	0.926
	average	0.644 ^#^	0.922	0.848
Vmax	systolic peak	0.998	1.000	0.998
	diastolic peak	0.927	1.000	0.997
Flow Rate	systolic peak	0.831	0.957	0.932
	diastolic peak	0.968	0.951	0.837
	average	0.281 ^n.s.^	0.785 *	0.866
Volume	systolic	0.956	0.989	0.963
	diastolic	0.958	0.972	0.923
	Net	0.293 ^n.s.^	0.794 *	0.879

**Legend:** ICC—interclass correlation, CSA—cross sectional area, Vmean—mean velocity inside the aqueduct of Sylvius, Vmax—highest velocity inside the aqueduct of Sylvius. All the ICCs were significant (*p* < 0.001; other significant *p*-values are noted as ^#^ *p* = 0.008; and * *p* = 0.002). The two non-significant ICCs are denoted with ^n.s.^ (*p* > 0.2).

**Table 3:** Parameter estimates of the GLM analyses.

Model Variables	B	Std. Error	t	95% Confidence Interval		Partial Eta Squared	Sig.
				Lower Bound	Upper Bound		
**CSA**							
age	0.026	0.009	2.969	0.009	0.043	0.059	0.004 *
sex	−0.597	0.185	−3.224	−0.963	−0.231	0.069	0.002 *
Jim	−0.083	0.211	−0.394	−0.500	0.334	0.001	0.694
Segment	0.103	0.211	0.487	−0.314	0.519	0.002	0.627
group	0.607	0.181	3.359	0.250	0.964	0.074	0.001 *
**Vmean systolic peak**							
age	0.028	0.017	1.590	−0.007	0.062	0.018	0.114
sex	−1.028	0.370	−2.776	−1.760	−0.296	0.052	0.006 *
Jim	0.032	0.421	0.077	−0.800	0.865	0.000	0.939
Segment	−0.044	0.421	−0.105	−0.877	0.788	0.000	0.916
group	0.695	0.361	1.924	−0.019	1.409	0.026	0.056
**Vmean diastolic peak**							
age	0.007	0.013	0.515	−0.019	0.033	0.002	0.608
sex	0.471	0.284	1.655	−0.091	1.033	0.019	0.100
Jim	−0.246	0.324	−0.759	−0.886	0.394	0.004	0.449
Segment	−0.100	0.324	−0.307	−0.739	0.540	0.001	0.759
group	−0.823	0.278	−2.966	−1.372	−0.275	0.059	0.004 *
**Average Vmean**							
age	−0.003	0.002	−1.091	−0.007	0.002	0.008	0.277
sex	−0.102	0.052	−1.976	−0.205	0.000	0.027	0.050
Jim	−0.044	0.059	−0.752	−0.161	0.072	0.004	0.453
Segment	−0.029	0.059	−0.491	−0.145	0.087	0.002	0.624
group	−0.051	0.050	−1.005	−0.151	0.049	0.007	0.316
**Vmax systolic peak**							
age	0.138	0.029	4.842	0.082	0.195	0.143	0.000 *
sex	−1.279	0.611	−2.095	−2.486	−0.072	0.030	0.038 *
Jim	−0.044	0.695	−0.063	−1.417	1.330	0.000	0.950
Segment	−0.071	0.695	−0.102	−1.444	1.303	0.000	0.919
group	0.562	0.596	0.943	−0.616	1.740	0.006	0.347
**Vmax diastolic peak**							
age	−0.049	0.019	−2.567	−0.086	−0.011	0.045	0.011 *
sex	0.892	0.406	2.196	0.089	1.695	0.033	0.030 *
Jim	0.003	0.462	0.006	−0.911	0.916	0.000	0.995
Segment	−0.030	0.462	−0.064	−0.943	0.884	0.000	0.949
group	−1.495	0.396	−3.772	−2.279	−0.712	0.092	0.000 *
**FR systolic peak**							
age	0.090	0.038	2.391	0.016	0.165	0.039	0.018 *
sex	−3.769	0.806	−4.676	−5.363	−2.176	0.134	0.000 *
Jim	−0.050	0.917	−0.055	−1.863	1.763	0.000	0.956
Segment	0.341	0.917	0.372	−1.472	2.154	0.001	0.711
group	2.341	0.787	2.976	0.786	3.896	0.059	0.003 *
**FR diastolic peak**							
age	−0.026	0.029	−0.874	−0.084	0.032	0.005	0.383
sex	2.087	0.629	3.318	0.844	3.331	0.072	0.001 *
Jim	0.041	0.716	0.057	−1.374	1.456	0.000	0.954
Segment	−0.366	0.716	−0.511	−1.781	1.049	0.002	0.610
group	−2.137	0.614	−3.481	−3.351	−0.923	0.079	0.001 *
**Average FR**							
age	−0.001	0.005	−0.263	−0.010	0.008	0.000	0.793
sex	−0.302	0.099	−3.047	−0.498	−0.106	0.062	0.003 *
Jim	0.052	0.113	0.458	−0.171	0.274	0.001	0.648
Segment	−0.002	0.113	−0.020	−0.225	0.221	0.000	0.984
group	0.151	0.097	1.558	−0.040	0.342	0.017	0.121
**Caudal volume**							
age	0.445	0.183	2.429	0.083	0.808	0.040	0.016 *
sex	−18.134	3.922	−4.623	−25.888	−10.379	0.132	0.000 *
Jim	0.509	4.462	0.114	−8.313	9.331	0.000	0.909
Segment	1.895	4.462	0.425	−6.927	10.716	0.001	0.672
group	10.495	3.828	2.742	2.927	18.062	0.051	0.007 *
**Cranial volume**							
age	−0.447	0.181	−2.467	−0.805	−0.089	0.041	0.015 *
sex	12.802	3.876	3.303	5.140	20.464	0.072	0.001 *
Jim	0.371	4.409	0.084	−8.346	9.088	0.000	0.933
Segment	−2.094	4.409	−0.475	−10.811	6.623	0.002	0.636
group	−7.856	3.782	−2.077	−15.334	−0.379	0.030	0.040 *
**Net volume**							
age	−0.002	0.076	−0.021	−0.151	0.148	0.000	0.984
sex	−5.332	1.619	−3.294	−8.532	−2.131	0.071	0.001 *
Jim	0.880	1.842	0.478	−2.761	4.520	0.002	0.634
Segment	−0.199	1.842	−0.108	−3.840	3.441	0.000	0.914
group	2.638	1.580	1.670	−0.485	5.761	0.019	0.097

CSA—cross sectional area, Vmean—mean velocity inside the aqueduct of Sylvius, Vmax—highest velocity inside the aqueduct of Sylvius, FR—flow rate. The variables used in each GLM are written in the first column. For each GLM, the dependent variable is written in bold and the independent ones are age, sex, software, and disease group. For the estimate calculation, the references for sex is male, for group is NC and for software is SPIN. Significant *p*-values (*p* < 0.05) are noted with an asterisk (*).

**Table 4:** PC-derived variables, compared among software packages using a general linear model covaried by age and sex. The mean values and standard deviations are grouped by software and by group; the Bonferroni-corrected *p*-values of the pair-wise comparisons are also reported.

PC-Derived Variable		Software			GLM Analysis *p*-Value			RM-ANOVA Analysis *p*-Value		
		Jim	Segment	SPIN	Jim vs. Segment	Jim vs. SPIN	Segment vs. SPIN	Jim vs. Segment	Jim vs. SPIN	Segment vs. SPIN
CSA (mm^2^)	average	2.59 ± 1.09	2.78 ± 1.22	2.68 ± 1.23	1	1	1	0.033 *	1.000	0.421
Vmean (cm/s)	systolic peak	5.64 ± 2.20	5.56 ± 2.23	5.6 ± 2.19	1	1	1	0.798	1.000	1.000
	diastolic peak	−4.43 ± 1.8	−4.28 ± 1.62	−4.18 ± 1.54	1	1	1	0.235	0.044 *	0.352
	average	0.19 ± 0.3	0.21 ± 0.29	0.23 ± 0.28	1	1	1	1.000	0.577	0.931
Vmax (cm/s)	systolic peak	9.65 ± 3.88	9.62 ± 3.84	9.70 ± 3.90	1	1	1	0.982	0.496	0.101
	diastolic peak	−7.01 ± 2.49	−7.04 ± 2.5	−7.02 ± 2.50	1	1	1	1.000	1.000	1.000
Flow rate (mL/min)	systolic peak	9.21 ± 5.22	9.60 ± 5.29	9.26 ± 5.26	1	1	1	0.149	1.000	0.089
	diastolic peak	−6.92 ± 3.67	−7.32 ± 4.1	−6.96 ± 3.87	1	1	1	0.064	1.000	0.082
	average	0.36 ± 0.59	0.3 ± 0.66	0.31 ± 0.47	1	1	1	1.000	1.000	1.000
volume (µL/cc)	caudal volume	31.89 ± 22.65	33.26 ± 23.51	32.04 ± 24.55	1	1	1	0.244	1.000	0.068
	cranial volume	−27.39 ± 24.61	−31.76 ± 32.71	−29.50 ± 27.37	1	1	1	0.103	1.000	0.106
	net	4.50 ± 5.46	1.50 ± 14.7	2.54 ± 3.88	1	1	1	1.000	1.000	1.000

**Legend:** GLM—General Linear Model, RM-ANOVA—Repeated Measure Analysis of Variance, CSA—cross sectional area, Vmean—mean velocity inside the aqueduct of Sylvius, Vmax—highest velocity inside the aqueduct of Sylvius. Significant *p*-values (<0.05) are noted with an asterisk (*).

**Table 5:** PC-derived variables, compared between groups using a general linear model covaried for age and sex. The pair-wise corrected comparisons are reported.

		Jim			Segment			SPIN		
		NC	MS	*p*-Value	NC	MS	*p*-Value	NC	MS	*p*-Value
CSA (mm^2^)		2.07 ± 0.87	2.93 ± 1.09	0.020 *	2.29 ± 1.15	3.09 ± 1.19	0.084	2.22 ± 1.24	2.96 ± 1.16	0.120
Vmean (cm/s)	systolic peak	5.11 ± 2.19	5.97 ± 2.18	0.377	4.96 ± 2.09	5.94 ± 2.26	0.277	4.96 ± 1.96	6.01 ± 2.26	0.211
	diastolic peak	−3.81 ± 2.19	−4.82 ± 1.42	0.082	−3.76 ± 2.06	−4.61 ± 1.18	0.11	−3.70 ± 1.98	−4.48 ± 1.12	0.113
	average	0.26 ± 0.27	0.15 ± 0.32	0.169	0.22 ± 0.34	0.19 ± 0.26	0.637	0.23 ± 0.23	0.24 ± 0.31	0.853
Vmax (cm/s)	systolic peak	8.90 ± 4.30	10.12 ± 3.58	0.614	8.83 ± 4.22	10.13 ± 3.56	0.567	8.91 ± 4.30	10.20 ± 3.61	0.610
	diastolic peak	−6.08 ± 2.85	−7.59 ± 2.06	0.036 *	−6.14 ± 2.91	−7.61 ± 2.06	0.045 *	−6.05 ± 2.92	−7.64 ± 2.01	0.035 *
Flow Rate (mL/min)	systolic peak	7.56 ± 6.37	9.83 ± 4.13	0.078	7.75 ± 6.38	10.26 ± 4.23	0.101	7.51 ± 6.62	9.93 ± 3.98	0.124
	diastolic peak	−5.54 ± 4.46	−7.49 ± 2.80	0.019 *	−5.68 ± 4.85	−8.12 ± 3.28	0.095	−5.18 ± 4.17	−7.73 ± 3.26	0.075
	average	0.30 ± 0.34	0.40 ± 0.71	0.811	0.12 ± 0.87	0.42 ± 0.47	0.214	0.18 ± 0.24	0.39 ± 0.55	0.209
Volume (µL/cc)	caudal	31.89 ± 22.65	47.37 ± 26.05	0.117	33.26 ± 23.51	48.76 ± 24.65	0.113	32.04 ± 24.55	46.44 ± 24.74	0.158
	cranial	−27.39 ± 24.61	−40.96 ± 19.17	0.105	−31.76 ± 32.71	−42.21 ± 19.77	0.375	−29.50 ± 27.37	−40.22 ± 20.01	0.301
	net	4.50 ± 5.46	6.41 ± 11.6	0.738	1.50 ± 14.7	6.54 ± 7.66	0.219	2.54 ± 3.88	6.21 ± 8.86	0.182

**Legend:** MS—multiple sclerosis, NC—normal controls, CSA—cross sectional area, Vmean—mean velocity inside the aqueduct of Sylvius, Vmax—highest velocity inside the aqueduct of Sylvius. Significant *p*-values (<0.05) are noted with an asterisk (*).

## Data Availability

The data presented in this study are available on request from the corresponding author.

## Supplementary Materials

The following are available online at https://www.mdpi.com/2075-4418/11/2/325/s1. Figure S1: Example of Regions of interest drawn for the Aqueduct of Sylvius (AoS) contours (red) and the background region (no-flow area, NFA) (light blue). The two ROIs are shown in the magnitude and phase images corresponding to the systolic peak; Figure S2: Effect of the background correction on the Aqueduct of Sylvius AoS mean velocity (Vmean): the Vmean curve over the cardiac cycle was computed using the three software packages with and without the background correction. The corrected (corr in the figure legend) and uncorrected (uncorr in the figure legend) Vmean are shown as solid and dotted lines, respectively.
