# Supplementary material for: Measuring Aqueduct of Sylvius Cerebrospinal Fluid Flow in Multiple Sclerosis Using Different Software
Source: Diagnostics (Basel). 2021 Feb 17;11(2):325. doi: 10.3390/diagnostics11020325 (PMC7923004; doi:10.3390/diagnostics11020325)
Supplement: Supplementary file 1 [file diagnostics-11-00325-s001.pdf]

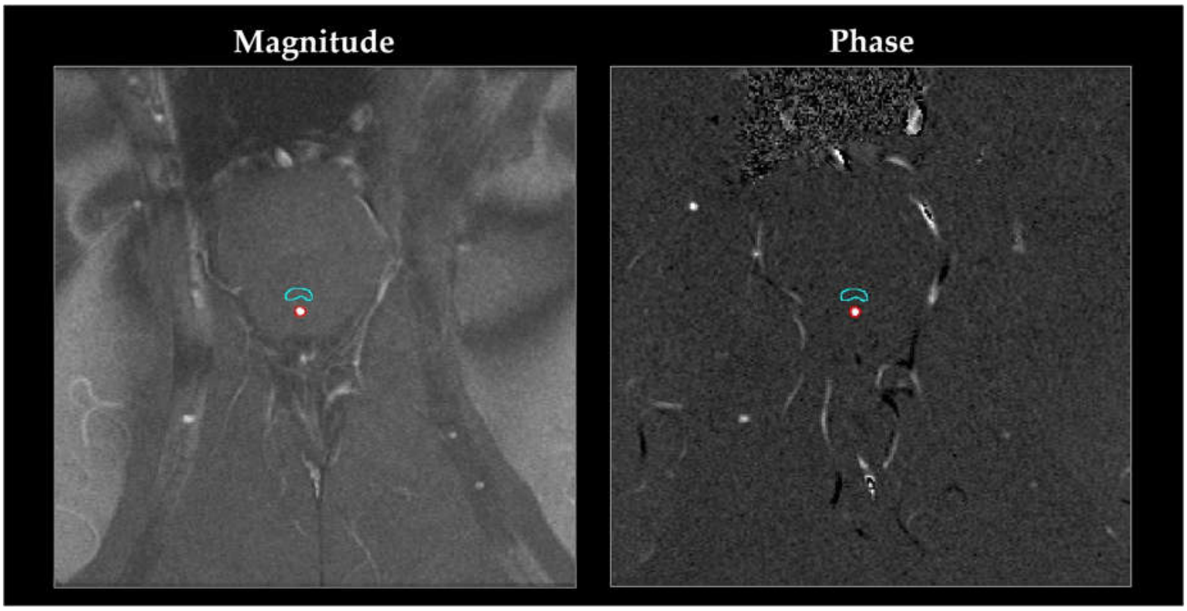

Figure S1: Example of Regions of interest drawn for the Aqueduct of Sylvius (AoS) contours (red) and the background region (no-flow area, NFA) (light blue). The two ROIs are shown in the magnitude and phase images corresponding to the systolic peak.

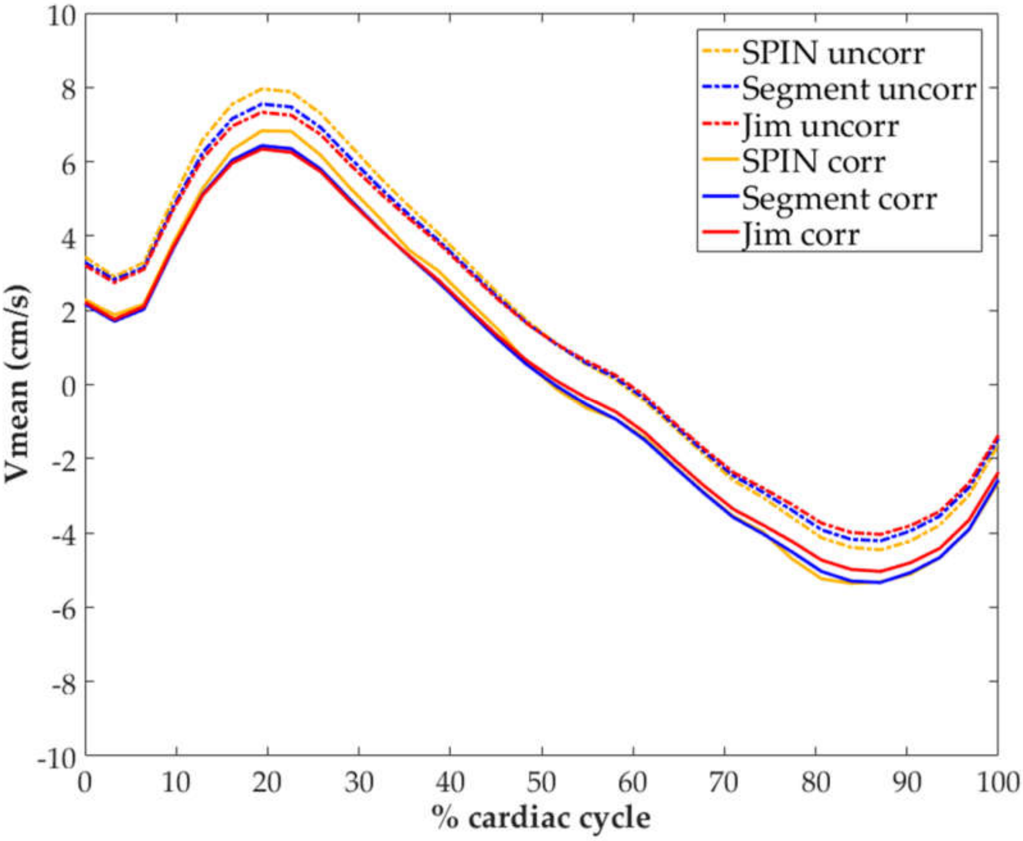

Figure S2: Effect of the background correction on the Aqueduct of Sylvius AoS mean velocity (Vmean): the Vmean curve over the cardiac cycle was computed using the three software pack-ages with and without the background correction. The corrected (corr in the figure legend) and uncorrected (uncorr in the figure legend) Vmean are shown as solid and dotted lines, respectively.
